# Association between non-traumatic vertebral fractures and adjacent discs degeneration: a cross-sectional study and literature review

**DOI:** 10.1186/s12891-020-03814-0

**Published:** 2020-11-27

**Authors:** Norihiko Takegami, Koji Akeda, Koichiro Murata, Junichi Yamada, Akihiro Sudo

**Affiliations:** Department of Orthopaedic Surgery, Mie University Graduate School of Medicine, 2-174 Edobashi, Tsu City, Mie 514-8507 Japan

**Keywords:** Vertebral fracture, Disc degeneration, Intradiscal vacuum phenomenon, Magnetic resonance imaging (MRI), Computed tomography (CT)

## Abstract

**Background:**

Previous clinical studies reported that thoracolumbar vertebral fractures (VFs) associated with high energy spine trauma cause adjacent intervertebral disc (IVD) degeneration; however, the effect of non-traumatic VFs on the progression of adjacent disc degeneration remains to be determined. The purpose of this study was to examine the association between non-traumatic VFs and degenerative changes of adjacent IVDs.

**Methods:**

Ninety-eight consecutive patients undergoing spinal surgery were included in this study. VFs were semi-quantitatively evaluated by lateral lumbar radiography. Five hundred eighty-eight vertebral bodies (from T12 to L5) and 486 discs (from T12/L1 to L4/L5) were analyzed. The degree of IVD degeneration was evaluated by magnetic resonance imaging (MRI) and classified into two groups according to Pfirrmann’s classification. Grades I, II and III were defined as the early stage of IVD degeneration and Grades IV and V as the advanced stage. Intradiscal vacuum phenomena (VPs) were evaluated by computed tomography. Adjacent IVDs were categorized according to the locations of VFs (superior, inferior, and bilateral). Associations between the presence of VFs and the extent of IVD degeneration or the presence of VPs were statistically analyzed.

**Results:**

IVDs adjacent to VFs were identified in 115 IVDs (31.1% of total; superior: 11.4%, bilateral: 8.6%, inferior: 11.1%). The presence of VFs was significantly associated with MRI grades of adjacent IVD degeneration (*P* < 0.01) and the prevalence of VPs within adjacent IVDs (*P* < 0.01). From logistic regression analysis, age, disc level, and VFs were independent related factors for disc degeneration (*P* < 0.05).

**Conclusion:**

This study showed that VFs were an independent related factor for adjacent disc degeneration and occurrence of intradiscal VPs. VFs may affect the micro-environment of adjacent IVDs, leading to disc degeneration and disc rupture.

## Background

Intervertebral discs (IVDs) consist of a central gelatinous nucleus pulposus and a surrounding fibrous annulus fibrosus (AF). IVDs are constrained within and connected to adjacent vertebral bodies by superior and inferior cartilaginous endplates (CEPs). Disc degeneration is considered to be caused by genetic predisposition, injury, aging, and environmental factors, or any combination thereof [1].

Blood flow to the vertebral bodies of the lumbar spine is abundantly supplied by the lumbar arteries, which are branches of the abdominal aorta [2]. IVDs are predominantly avascular and aneural tissues that exchange nutrients and metabolites primarily by diffusion to and from micro-vessels in the CEP and outer AF [3, 4]. The restricted transport and low cellularity of the discs limit repair. Therefore, endplate sclerosis, or an ischemic vertebra, is considered to be one of the factors responsible for IVD degeneration [1, 5].

Previous clinical studies reported that a thoracolumbar burst fracture with high energy spine trauma caused disc degeneration, and that, importantly, disc degeneration occurred at a level adjacent to the fractured vertebra [6–11]. One multicenter cohort study recently reported that progression of adjacent disc degeneration was observed at 6 months after osteoporotic VFs [12]. However, further study is needed to determine the association between non-traumatic VFs, including osteoporotic fractures, and the progression of disc degeneration adjacent to VFs.

The purpose of this cross-sectional population study was to examine the effect of non-traumatic VFs on degenerative changes of adjacent IVDs using magnetic resonance imaging (MRI) and computed tomography (CT) analyses.

## Methods

### Subject

This IRB-approved retrospective study was conducted on spinal CT images of 98 consecutive patients (50 males and 48 females) undergoing spinal surgery (Table 1).

**Table 1:** Patient characteristics

Group	Subjects (males/females)	Average age (years)*
VF-negative	50 (25/25)	60.6 (23–87)
VF-positive	48 (25/23)	75.4 (56–90)
Total	98 (50/48)	68.2 (23–90)

*VF* Vertebral fracture. The numbar in the parenthesis in the average age column indicates the age range of subjects. *P < 0.01 between VF-negative and VF-positive groups

The overall average age of the patients was 68.2 years-old (range 23–90). The clinical diagnoses of the patients were as follows: 74 lumbar spinal stenosis, 15 lumbar disc herniation, 6 cervical spinal diseases, and 3 others. Patients with VFs caused by high energy trauma were excluded from this study.

### Morphological classification of VFs

VFs were evaluated using lateral lumbar radiographs in all patients; 588 vertebral bodies from T12 to L5 were analyzed. VF deformities were classified into three groups (wedge, biconcave, or crush) using a semi-quantitative technique [13].

### Classification of disc degeneration

MRI was performed in 74 patients. A total of 370 discs from T12/L1 to L4/L5 were analyzed with MRI. The degree of disc degeneration was evaluated with sagittal T2-weighted lumbar MRI, and graded according to Pfirrmann’s classification from Grades I to V [14]. Grades I, II and III were defined as the early stage of IVD degeneration and Grades IV and V as the advanced stage.

### Diagnosis of vacuum phenomena (VPs)

Multi-detector CT (MDCT) (slice increment: 1.0 mm, slice thickness: 1.0 mm; Asteion TSX-021B, Toshiba Medical Systems Co., Otawara, Tochigi, Japan) was performed for all patients. A total of 486 discs from T12/L1 to L4/L5 were analyzed with MDCT. Four discs were excluded, because the T12/L1 disc was outside the range of CT analysis in four patients. Intradiscal VPs were evaluated by the presence of areas of gaseous radiolucency using MDCT imaging and those shapes were classified using sagittal imaging as previously reported [15]. In short, VP shapes were categorized according to three classifications: spot, linear, and island. A spot-type VP was defined as a point-like VP less than 2 mm in diameter. A linear-type rupture was defined as a radiating VP whose width was less than 2 mm. An island-type rupture was defined as a VP forming a wide cleft (> 2 mm).

### Categorization of IVDs

**Fig. 1:**
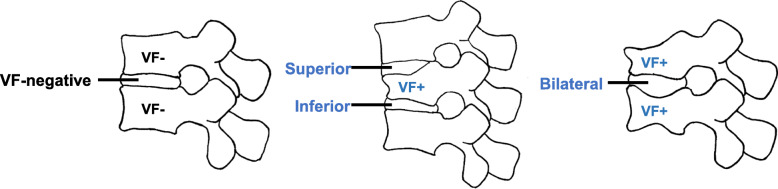
Categorization of intervertebral discs (IVDs). IVDs were categorized according to the locations of adjacent vertebral fractures (VFs). The VF-negative (VF-) group was defined as those IVDs having no fracture in an adjacent vertebral body. The VF-positive (VF+) group was defined as those IVDs having a fracture in an adjacent vertebral body. The VF-positive group was further classified into three subgroups: those IVDs superior to the VF (superior), those IVDs inferior to the VF (inferior), and those IVDs located between VFs (bilateral)

IVDs were categorized according to the locations of adjacent VFs. The control (VF-negative) group was defined as those IVDs having no fracture in an adjacent vertebral body. The VF-positive group was defined as those IVDs having fractures in the adjacent vertebral body. Furthermore, the VF-positive group was further classified into three subgroups: those with IVDs superior to a VF (superior group), those with IVDs inferior to a VF (inferior group), and those with IVDs located between VFs (bilateral group) (Fig. 1).

### Statistical analysis

The morphological classification of VFs, the classification of disc degeneration and the diagnosis of VPs were evaluated by a single observer who was blinded to the categorization of the IVD groups. The Chi-square test and Student’s t-tests were used to compare the differences in gender and age between patients with VFs and those without VFs. The differences in the percentage of advanced stage of disc degeneration (MRI) or the prevalence of VP among the categorization of IVDs according to the locations of adjacent VFs were statistically assessed by a chi-square test followed by post hoc multiple comparisons using the Bonferroni method [15]. The post hoc test was performed to assess the probability values for each combination of independent category levels by using a Bonferroni correction to control for type I error inflation [16, 17]. Significance was determined by Chi-square test with post-hoc analysis by cellwise adjusted residual analysis in two-way contingency tables according to Garcia-Perez [16–18]. Post-hoc testing was performed with adjusted standardized residual analysis with an eight-fold Bonferroni-adjusted *p*-value (*p* < 0.006) or sixteen-fold Bonferroni-adjusted p-value (*p* < 0.003) [19].

The association between disc degeneration and the prevalence of an adjacent VF was evaluated by multiple logistic regression. Factors included in the multivariate model were age, gender, disc level, and adjacent VF(s). All the statistical analyses were performed using IBM SPSS Statistics (IBM Japan, Tokyo or IBM Corp., Armonk, NY, USA).

## Results

### Patient characteristics

**Fig. 2:**
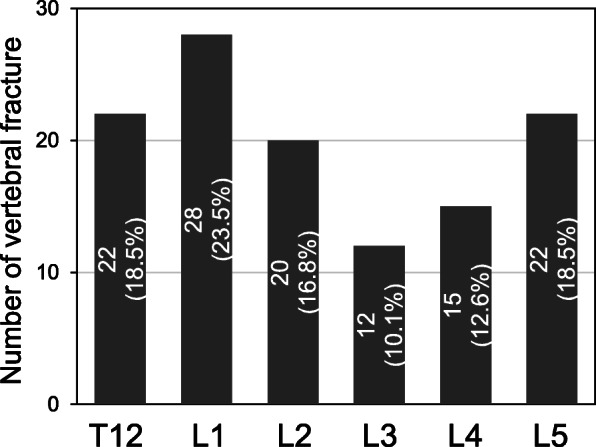
Number of vertebral fractures (VFs) at different vertebral levels. VFs were evaluated by lateral lumbar radiography. VFs were identified in 119 vertebrae (20.4%) of the 584 vertebral bodies analyzed

VFs were identified in 119 vertebrae (20.4%) of the 584 vertebral bodies analyzed (wedge type: 51.3%; biconcave type: 37.8%; crush type: 10.9%). The number of VFs was highest in the L1 vertebral body and lowest in the L3 (n [% of total VFs]: T12: 22 [18.5%]; L1: 28 [23.5%]; L2: 20 [16.8%]; L3: 12 [10.1%]; L4: 15 [12.6%]; L5: 22 [18.5%]) (Fig. 2). IVDs adjacent to VFs were identified in 115 IVDs (31.1%) of the 370 IVDs analyzed by MRI (superior: 42 [36.5%], bilateral: 32 [27.8%], inferior: 41 [35.7%]). IVDs adjacent to VFs were identified in 147 IVDs (30.2%) of the 486 IVDs analyzed by CT analysis (superior: 51 [34.7%], bilateral: 45 [30.6%], inferior: 51 [34.7%]).

### Association between VFs and disc degeneration

**Fig. 3:**
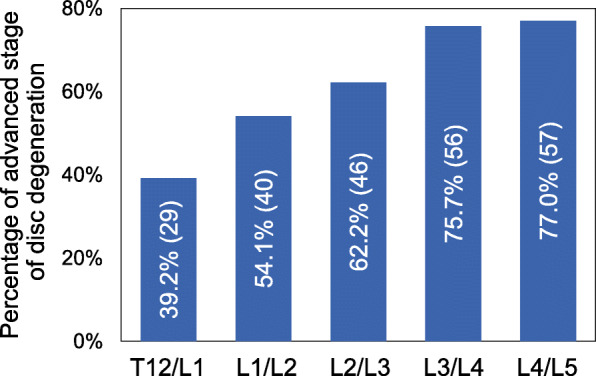
Percentage of advanced stage disc degeneration at different intervertebral disc (IVD) levels. The degree of disc degeneration was evaluated by sagittal T2-weighted lumbar magnetic resonance imaging (MRI) and was graded according to Pfirrmann’s classification [14] from Grades I to V. Grades I, II and III were defined as early stage IVD degeneration and Grades IV and V as advanced stage

Of all the discs analyzed by MRI (370 discs), 228 IVDs (61.6%) were classified as advanced degenerative stage, appearing most frequently in the L4/L5 disc and least frequently in the T12/L1 disc (T12/L1: 39.2%, L1/L2: 54.1%, L2/L3: 62.2%, L3/L4: 75.7%, L4/L5: 77.0%) (Fig. 3).

**Fig. 4:**
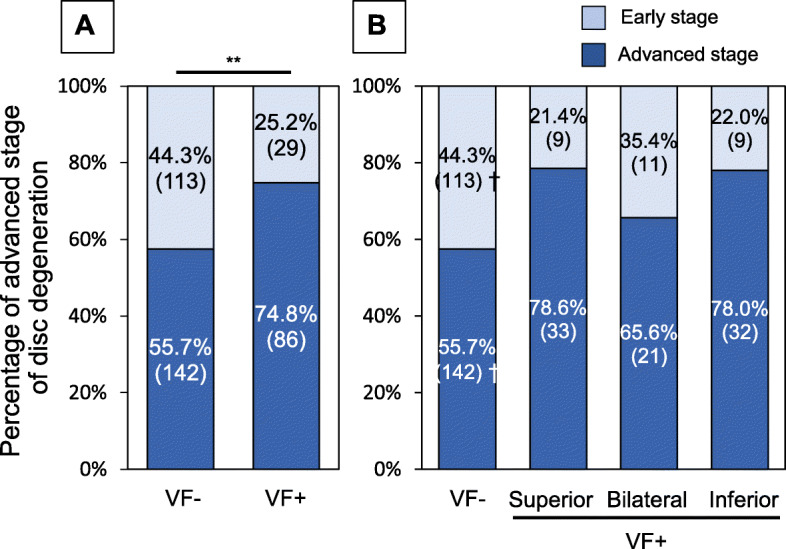
Percentage of advanced stage disc degeneration adjacent to vertebral fractures (VFs). **a** The percentage of advanced stage disc degeneration evaluated by magnetic resonance imaging (MRI) was compared between VF-negative (VF-) and VF-positive (VF+) groups. **P < 0.01 (by chi-square test). **b** The VF+ group was classified into the following three subgroups: 1. IVDs superior to VFs (superior group), 2. IVDs inferior to VFs (inferior group), and 3. IVDs located between VFs (bilateral group). † *P* < 0.006 (by Bonferroni correction)

A chi-square test showed a significant association between the presence of VFs and MRI grades of IVD degeneration adjacent to VFs (*P* < 0.01) (Fig. 4a). The results of a post-hoc test showed that the numbers of IVDs with advanced stage degeneration were significantly lower than expected in the VF-negative group (*P* < 0.006) (Fig. 4b). However, those with advanced stage degeneration were not significantly higher than expected in the IVDs of superior, bilateral, and inferior groups (superior: 78.6%, bilateral: 65.6%, inferior: 78.0%) (Fig. 4b).

There were no significant differences in the prevalence of advanced stage IVD degeneration among the deformity types of adjacent VF by chi-square test (wedge: 77%; biconcave: 76%; crush: 100%).

**Fig. 5:**
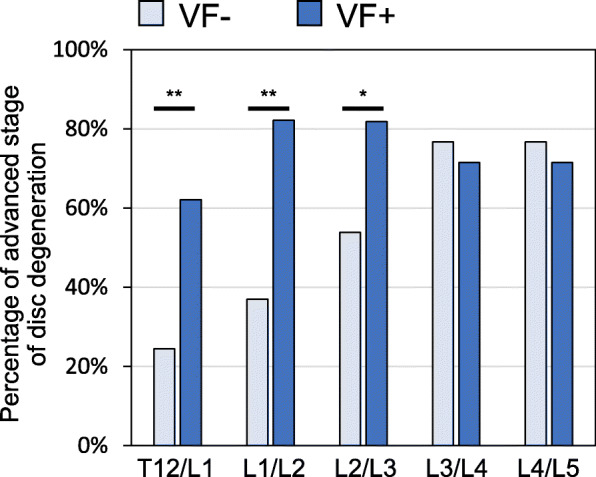
Percentage of advanced stage disc degeneration adjacent to vertebral fractures (VFs) at different intervertebral disc levels. The percentages of advanced stage disc degeneration in T12/L1, L1/L2 and L2/L3 in the VF-positive group were significantly higher than those in the VF-negative group. However, there was no significant difference in the percentages of advanced stage disc degeneration between the VF-positive group and the VF-negative group in L3/L4 and L4/L5. *P < 0.05, **P < 0.01 between VF- and VF+

When the proportion of disc degeneration was analyzed by disc level, the percentages of advanced stage disc degeneration in T12/L1, L1/L2 and L2/L3 in the VF-positive group were significantly higher than those in the VF-negative group (Fig. 5). However, there were no significant differences in the percentages of advanced stage disc degeneration between the VF-positive group and the VF-negative group in L3/L4 and L4/L5.

The results of logistic regression analysis showed that age, disc level, and adjacent VFs were independent factors that were significantly associated with the MRI-grade of IVD degeneration (Table 2).

**Table 2:** Logistic regression analysis of significant related factors for advanced stage of disc degeneration and intradiscal vacuum phenomenon

		odds ratio (95%CI)	***p*** value
**DD**	**age**	1.050 (1.029–1.071)	< 0.001
	**disc level**	1.687 (1.412–2.017)	< 0.001
	**adjacent VF**	1.906 (1.079–3.368)	0.026
**VP**	**age**	1.054 (1.034–1.076)	< 0.001
	**disc level**	1.513 (1.298–1.764)	< 0.001
	**adjacent VF**	2.476 (1.553–3.949)	< 0.001

*DD* Disc degeneration, *VP* Vacuum phenomenon, *VF* Vertebral fracture

### Association between VFs and intradiscal VPs

**Fig. 6:**
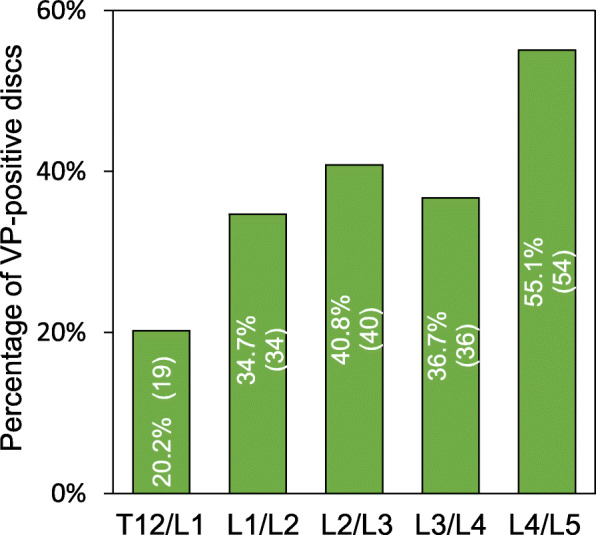
Number of vacuum phenomenon (VP)-positive discs at different intervertebral disc levels. Intradiscal VP was evaluated by the presence of areas of gaseous radiolucency using multi-detector computed tomography (MDCT) imaging [15]. VPs were found in 226 IVDs (46.5%) of the 486 IVDs analyzed

VPs were found in 226 IVDs (46.5%) of the 486 IVDs analyzed. The number of discs with a VP was highest in the L4/5 level (55.1%). At other levels, VPs were found in 34–41% of the discs, with the exception of the T12/L1 level, where a VP was found in 20.2% of IVDs (Fig. 6).

**Fig. 7:**
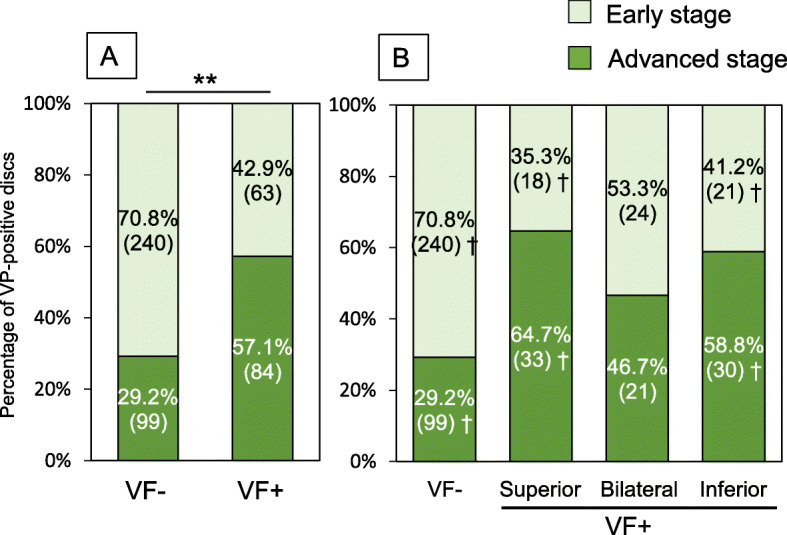
Percentage of vacuum phenomenon (VP)-positive discs adjacent to vertebral fractures (VFs). **a** Percentage of VP-positive discs between VF-negative (VF-) and VF-positive (VF+) groups. **P < 0.01 (by chi-square test). **b** The VF+ group was classified into the following three subgroups: 1. IVDs superior to VFs (superior group), 2. IVDs inferior to VFs (inferior group), and 3. IVDs located between VFs (bilateral group). † *P* < 0.006 (by Bonferroni correction)

A chi-square test showed a significant association between the presence of VFs and VPs within IVDs adjacent to a VF (*P* < 0.01) (Fig. 7a). The results of a post-hoc test showed that the numbers of IVDs with VPs were significantly lower than expected in the VF-negative group (29.2%) (*P* < 0.006), and significantly higher than expected in superior and inferior groups (superior: 64.7%, inferior: 58.8%) (*P* < 0.006) (Fig. 7b). In the VP shape analysis, the number of IVDs with an island shape VP was significantly lower than expected in the VF-negative group (*P* < 0.003), and significantly higher than expected in the superior group (*P* < 0.0006) (Table 3).

**Table 3:** Association between the location of intervertebral discs (IVDs) adjacent to vertebral fractures (VFs) and the shape of intradiscal vacuum phenomena (VPs)

IVD location		VP shape				Total
		VP (−)	Spot	Linear	Island	
VF (−)	Count (% raw)	240** (70.8)	18 (5.3)	29 (8.6)	52* (15.3)	339
	Exp. Count	**221.4**	25.8	37.0	**64.9**	
	Corrected *P*-value	**< 0.0006**	0.004	0.012	**< 0.003**	
Superior	Count (% raw)	18** (35.3)	9 (17.6)	5 (9.8)	19** (37.3)	51
	Exp. Count	**31.8**	3.9	5.6	**9.8**	
	Corrected *P*-value	**< 0.0006**	0.004	0.790	**< 0.0006**	
Bilateral	Count (% raw)	24 (53.3)	5 (11.1)	10 (22.2)	6 (13.3)	45
	Exp. Count	28.1	3.4	4.9	8.6	
	Corrected *P*-value	0.190	0.353	0.011	0.299	
Inferior	Count (% raw)	21* (41.2)	5 (9.8)	9 (17.6)	16 (31.4)	51
	Exp. Count	**31.8**	3.9	5.6	9.8	
	Corrected *P*-value	**< 0.003**	0.533	0.103	0.019	
	Total	303	37	53	93	486

A chi-square test showed a significant association between the deformity type of VF and the prevalence of a VP within IVDs adjacent to the VF (*P* < 0.05). There was a tendency for the percentage of VPs to be lower in IVDs adjacent to wedge type VFs (52%). VPs were identified in all IVDs adjacent to crush type VFs (*n* = 7).

The results of a contingency-table test are presented. Intervertebral discs (IVDs) were categorized according to the locations of adjacent vertebral fractures (VFs). Percentages of the raw marginal total (% raw) are in parentheses. VF (−): no VF group; VP (−); no vacuum phenomena group; Exp. Count: expected count. *P < 0.003, **P < 0.0006 (by Bonferroni correction). The cells with counts above or below the expected count with statistical significance are shown in bold.

**Fig. 8:**
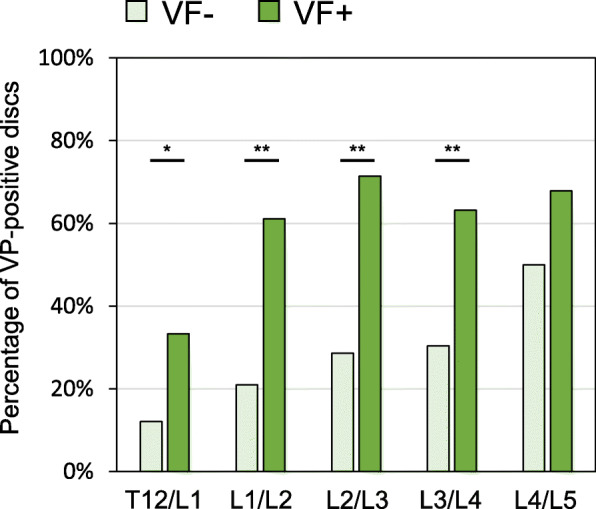
Percentage of vacuum phenomenon (VP)-positive discs adjacent to vertebral fractures (VFs) at different intervertebral disc levels. The percentages of VP-positive discs in T12/L1, L1/L2, L2/L3 and L3/L4 in the VF-positive group were significantly higher than those in the VF-negative group. *P < 0.05, **P < 0.01 between VF- and VF+ groups

When the prevalence of intradiscal VPs was analyzed by disc level, the percentages of VP-positive discs in T12/L1, L1/L2, L2/L3 and L3/L4 in the VF-positive group were significantly higher than those in the VF-negative group (Fig. 8). However, there was no significant difference in the percentages of VP-positive discs between the VF-positive and -negative groups in the L4/L5 level.

A logistic regression analysis revealed that age, disc level, and adjacent vertebral fracture were independent factors that were significantly associated with the presence of intradiscal VPs (Table 2).

## Discussion

We conducted a cross-sectional retrospective study of consecutive patients undergoing spinal surgery to evaluate the association between non-traumatic VFs and IVD degeneration adjacent to VFs. VFs were significantly associated with MRI grades of IVD degeneration and the presence of intradiscal VPs of discs adjacent to VFs. Logistic regression analysis showed that VF was one of the independent related factors for adjacent disc degeneration.

Eight clinical studies have evaluated the association between thoracolumbar VFs with spine trauma and adjacent disc degeneration (Table 4). Among these, two studies focused on degenerative IVDs adjacent to a VF in children or young patients who were treated conservatively [6, 20]. Kerttula et al. investigated the occurrence of disc degeneration by MRI in young patients (average-age: 15.5 years) with a history of wedge type VFs [6]. They concluded that wedge type VFs, especially with endplate injury, in young people were significantly associated with the occurrence of disc degeneration [6]. Later, Moller et al. [20] evaluated whether VFs in children (average-age: 12 years) are a risk factor for adjacent IVD degeneration. They used MRI and the Oner classification scheme [21], which mainly classifies morphological changes of IVDs and endplate injuries, and reported no significant association between stable VFs and adjacent disc degeneration [20]. Because of differences in MRI assessments and type of injuries (Table 4), the relationship between VFs and adjacent disc degeneration in young people and children remains controversial. The other six clinical studies had evaluated disc degeneration following thoracolumbar VFs (AO classification [25]: type A1–4) treated with spine surgeries (Table 4). Posterior pedicle screw fixation was performed in four studies [7–9, 22] and instrumented kyphoplasty in two studies [10, 11]. Among six clinical studies, adjacent disc degeneration was evaluated by MRI in five studies [7–10, 22], and by radiograph in one study [11]. These studies have shown that adjacent disc degeneration had significantly progressed at 9 to 32 months after thoracolumbar burst fractures in comparison with those at the time of injury, except for one study reported by Verlaan et al. [22]. Three studies [8, 10, 11] have reported that disc degeneration was predominantly found at the superior adjacent disc. On the other hand, Sander et al. [9] reported that the disc degradation was identified both at superior and inferior adjacent discs after traumatic VFs. Toyone et al. [7] also showed that adjacent disc degeneration had progressed at 2 years after burst fractures; however, the degeneration of superior and inferior adjacent discs were not separately analyzed. Lastly, Verlaan et al. [22] reported that 10.5% of the superior adjacent discs and 15.8% of inferior adjacent discs showed progression of degeneration at 12 to 18 months after trauma. However, they concluded that no statistically significant progression in adjacent disc degeneration was found for superior or inferior discs.

**Table 4:** Association between thoracolumbar vertebral fractures with spine trauma and the adjacent disc degeneration

Author	Year	Subjects	Age (averaged)	Design	Follow-up	Type of injury	Treatment	MRI Assessment
Kerttula [6]	2000	14	15.5 [8.8–20.8]	Retrospective study	3.8 Y	Wedge-type (14)	Conservative	Decrease in T2 signal intensity
Moller [20]	2007	20	12 [7–16]	Observational cohort study	40 Y	Stable (18), Denis type B (2)	Conservative	Oner classification Scheme [21]
Verlaan [22]	2013	20	42 [18–74]	Prospective trial	12 to 18 M	AO: A3 (20), A4 (1)	PS fixation	Pfirrmann classification [14]
Toyone [7]	2013	12	38 [14–59]	Prospective consecutive series	10 Y	AO: A3 (12)	PS fixation	Borenstein’s report (score: 0–3) [23]
Wang [8]	2013	26	39.6 [21–54]	Retrospective study	9–12 M	AO: A3 (26)	PS fixation	Pfirrmann classification [14]
Sander [9]	2014	27	37.5 [16–59]	Retrospective study	1 Y	AO: A1 (5), A2 (14), A3 (8)	PS fixation	Original classification (Grade 0–3)
Noriega [10]	2016	20	50.7 [45–56]	Retrospective study	32 M	AO: A1 (10), A3 (10)	IKP	Diffusion-weighted MR imaging
Descamps [11]	2019	93	54 [18–83]	Retrospective study	26.7 M	AO: A1 (54), A2 (5), A3 (34)	IKP	Radiograph UCLA Grading Scale [24]

Numbers in the brackets indicate the range of age. Number in the parentheses indicates the number of subjects

*Y* Years, *M* Months, *PS fixation* Pedicle screw fixation, *AO* AO classification [19], *IKP* Instrumented kyphoplasty

*OVF* Osteoporotic vertebral fracture

In contrast to these previous studies, the results of the current study showed a significant association between non-traumatic VFs and disc degeneration, at both superior and inferior adjacent levels, in a relatively older population.

Rahmani et al. have recently evaluated whether endplate fracture (injury) and adjacent disc degeneration have a significant association with the occurrence of delayed union following osteoporotic VFs for 139 consecutive patients (average age: 79 years-old) who were treated conservatively [12]. They also evaluated signal changes of adjacent IVDs in MR T2-weighted images at enrollment and at 6 months follow-up based on a modified Pfirrmann grading system and reported that adjacent cranial disc degeneration had significantly progressed at 6 months post-injury. This suggests the possibility that disc degeneration would progress in the relatively short term after osteoporotic VFs.

Next, to evaluate the effect of spinal levels on disc degeneration, the relationship between VFs and disc degeneration at different IVD levels was assessed (Fig. 5). Although there was no significant difference in the percentages of advanced stage of disc degeneration between VF-positive and VF-negative groups in L3/L4 and L4/L5, those percentages in T12/L1, L1/L2 and L2/L3 discs, which would be expected to have less degeneration than lower lumbar levels [26], were significantly higher in the VF-positive group than in the VF-negative group. This suggests that the effect of VFs on adjacent disc degeneration would be more pronounced at upper lumbar levels than those at middle/lower lumbar levels. There is also evidence to support this suggestion from cadaveric studies [27–31]. Dolan et al. [27] performed a mechanical and morphological study to evaluate how spinal level influences disc degeneration arising from endplate fracture. They reported that the effects of vertebral endplate fracture on disc mechanical function, and specifically on disc decompression, were greater at thoracic and upper lumbar levels than at lower lumbar levels.

In addition, we evaluated the association between VFs and the presence of adjacent intradiscal VPs. Intradiscal VPs refer to the radiographic appearance of a lucency caused by the presence of gas, usually found in the lumbar region [32, 33]; this is one of the characteristics of IVD degeneration [15, 34]. Murata and colleagues showed that the presence of intradiscal VPs is associated with the MRI-grade of disc degeneration and radiographic disc height narrowing [15]. The results of the current study showed that the incidence of VPs, especially the island type, in the VF-positive group was significantly higher than that in the VF-negative group; this suggests that VFs have an impact, not only on the extent of MRI-graded disc degeneration, but also on the intradiscal ruptures evaluated by CT imaging as intradiscal VPs.

The results of the analysis of intradiscal VPs by disc level were nearly identical to those of MRI-graded disc degeneration. Lafforgue and colleagues reported that VPs were grouped into collapse-related VPs and degenerative VPs [35]. They reported that collapse-related VPs, which were secondary to vertebral collapse, were located mainly in the thoracolumbar junction. Degenerative VPs, which were the result of disc degeneration, were located in lower lumbar discs. Therefore, in the current study, we speculate that intradiscal VPs in upper lumbar levels would be mainly attributed to VFs (vertebral collapse).

According to the results of logistic regression analyses, age, disc level, and adjacent vertebral fracture were independent related factors for disc degeneration and intradiscal VPs (Table 2). It is well known that being elderly and lower disc level were significant related factors for disc degeneration [26, 36, 37]. The current study showed evidence that VFs are also an independent related factor for adjacent disc degeneration for the population with non-traumatic VFs.

The following three patho-mechanisms are involved in the occurrence of adjacent disc degeneration. First, endplate and IVD injuries directly caused by VFs promote the progression of disc degeneration. Fujiwara and colleagues reported that endplate injuries were observed in 61%, and IVD lesions in 60% of patients with an acute, single osteoporotic VF [38]. Second, the progression of vertebral collapse is thought to cause impaired blood flow in vertebral bodies, to reduce blood flow and nutrient supply to the disc, and to cause disc degeneration [1, 5]. Imanishi and colleagues recently showed using a rabbit lumbar artery ligation model that ischemia of lumbar vertebrae initiated degenerative changes in IVDs [4]. Therefore, an ischemic vertebra is considered to be one of the important factors responsible for IVD degeneration. Third, mechanical stress is also involved in the progression of adjacent disc degeneration. Dolan et al. reported that vertebral endplate fracture reduced nucleus pressure and created abnormal stress distributions in the adjacent IVD, increasing the risk of internal disruption and degeneration [27]. Interestingly, Stefanakis et al. and Zehra et al. performed mechanical and morphological studies to determine whether high gradients of compressive stress within the IVD are associated with progressive disc degeneration [29, 31]. They reported that as the grade of disc degeneration increased, nucleus pressure decreased. However, stress gradients (concentration) in the annulus increased.

A limitation of this study is that most of the subjects were patients who had been given pre-operative radiographs, CT, and MRI for elective spinal surgeries. Therefore, MRI grading of IVDs and percentage of intradiscal VPs would be much higher than those within a general population [26]. Another limitation is that VFs in our study excluded those caused by high energy trauma. Although the evaluation of osteoporosis was not performed in this study, most VFs in the subjects of this study would be osteoporotic VFs. Thirdly, the other risk factors associated with VFs, such as obesity and physical activity, and the clinical outcome including the subject’s low back pain have not been evaluated in this study. Further study would be needed to evaluate the risk factors of adjacent disc degeneration following VFs, and the association of adjacent disc degeneration and clinical outcomes.

## Conclusions

This study showed that non-traumatic VFs are an independent related factor for adjacent disc degeneration and the occurrence of intradiscal VPs at the corresponding level. From the results of the current study, we speculate that VFs may affect the micro-environment of adjacent IVDs, leading to progression of disc degeneration and disc ruptures. Therefore, careful follow-up is necessary even for non-traumatic VFs (mostly osteoporotic VFs) and proper treatment, including surgical intervention, of VFs may prevent the progression of disc degeneration.

## Data Availability

The datasets used and analyzed during the current study are available from the corresponding author at a reasonable request.

## Abbreviations

VF: Vertebral fracture
IVD: Intervertebral disc
AF: Annulus fibrosus
CEP: Cartilaginous endplate
CT: Computed tomography
MDCT: Multi-detector CT
VP: Vacuum phenomenon
MRI: Magnetic resonance imaging
